# A Full Index List of Contributors

**Published:** 1880-12

**Authors:** 


A Full Index List of Contributors, *and Title Pages*, for volume one will be published with the January issue of the Journal. When the importance and variety of the information contained in its six hundred pages of reading matter are considered we feel warranted in saying that volume one of the *Independent Practitioner*, is the most valuable professional work that has been published for the small sum of two dollars.

